# Validation of shear wave elastography for assessing myocardial fibrosis in patients with end-stage heart failure

**DOI:** 10.1093/ehjci/jeaf375

**Published:** 2026-01-06

**Authors:** Henrik B Dukefoss, Kristoffer Andresen, Markus B Harbo, Nina E Hasselberg, Herman Grødem, Truls Korsæth, Henrik Romundstad, Kjell Kristoffer W Moe, Lasse Løvstakken, Einar Gude, Tuva B Dahl, Johannes L Bjørnstad, Bente Halvorsen, Arnt E Fiane, Kaspar Broch, Ivar Sjaastad, Thor Edvardsen, Emil K S Espe

**Affiliations:** Institute for Experimental Medical Research, Oslo University Hospital and University of Oslo, Kirkeveien 166, Oslo 0450, Oslo, Norway; Faculty of Medicine, University of Oslo, Oslo, Norway; ProCardio Center for Innovation, Department of Cardiology, Oslo University Hospital, Oslo, Norway; Institute for Experimental Medical Research, Oslo University Hospital and University of Oslo, Kirkeveien 166, Oslo 0450, Oslo, Norway; ProCardio Center for Innovation, Department of Cardiology, Oslo University Hospital, Oslo, Norway; Institute for Experimental Medical Research, Oslo University Hospital and University of Oslo, Kirkeveien 166, Oslo 0450, Oslo, Norway; Institute for Experimental Medical Research, Oslo University Hospital and University of Oslo, Kirkeveien 166, Oslo 0450, Oslo, Norway; Institute for Experimental Medical Research, Oslo University Hospital and University of Oslo, Kirkeveien 166, Oslo 0450, Oslo, Norway; Institute for Experimental Medical Research, Oslo University Hospital and University of Oslo, Kirkeveien 166, Oslo 0450, Oslo, Norway; Norwegian University of Science and Technology, and St Olavs University Hospital, Trondheim, Norway; ProCardio Center for Innovation, Department of Cardiology, Oslo University Hospital, Oslo, Norway; Research Institute of Internal Medicine, Oslo University Hospital Rikshospitalet, Oslo, Norway; Faculty of Medicine, University of Oslo, Oslo, Norway; Department of Cardiothoracic Surgery, Rikshospitalet, Oslo, Norway; Faculty of Medicine, University of Oslo, Oslo, Norway; Research Institute of Internal Medicine, Oslo University Hospital Rikshospitalet, Oslo, Norway; Department of Cardiothoracic Surgery, Rikshospitalet, Oslo, Norway; Faculty of Medicine, University of Oslo, Oslo, Norway; ProCardio Center for Innovation, Department of Cardiology, Oslo University Hospital, Oslo, Norway; Institute for Experimental Medical Research, Oslo University Hospital and University of Oslo, Kirkeveien 166, Oslo 0450, Oslo, Norway; Faculty of Medicine, University of Oslo, Oslo, Norway; ProCardio Center for Innovation, Department of Cardiology, Oslo University Hospital, Oslo, Norway; Institute for Experimental Medical Research, Oslo University Hospital and University of Oslo, Kirkeveien 166, Oslo 0450, Oslo, Norway

**Keywords:** shear wave elastography, myocardial fibrosis, heart failure, myocardial stiffness, high-frame-rate echocardiography, collagen volume fraction

## Abstract

**Aims:**

Myocardial fibrosis plays a crucial role in the pathophysiology of heart failure, increases myocardial stiffness, impairs diastolic function and is associated with adverse outcomes. Shear wave elastography (SWE) uses high frame rate echocardiography to assess shear waves in the myocardium. This technique may permit assessment of myocardial stiffness by measuring the propagation speed of myocardial shear waves. We aimed to validate the ability of SWE to assess the degree of myocardial fibrosis in patients with end-stage heart failure.

**Methods and results:**

We performed high frame rate echocardiography in 16 heart failure patients who were listed for heart transplantation and 16 age- and sex-matched healthy control subjects. Naturally occurring shear waves triggered by mitral valve closure (MVC) and aortic valve closure (AVC) were analyzed by tissue Doppler imaging in the interventricular septum. Septal shear wave velocities were compared with septal collagen volume fraction (CVF) in explanted hearts. Mean septal CVF in cardiac explants was 17.1 ± 7.6%. AVC and MVC wave velocities were associated with septal CVF (Spearman’s correlation of mean septal values: *ρ* = 0.75, *P* = 0.02 and *ρ* = 0.65, *P* = 0.03, respectively). Similar results were observed in linear mixed-effects regression analysis by septal region (AVC wave: *β* = 2.2, 95% CI [0.7, 3.8], *P* = 0.004; MVC wave: *β* = 1.5, 95% CI [0.4, 2.5], *P* = 0.005). Shear waves could be measured in most patients but were limited by patient factors such as prosthetic valves and left ventricular assist devices.

**Conclusion:**

Shear wave velocities from SWE correlate with CVF. SWE therefore hold promise as a novel non-invasive method for assessing myocardial fibrosis.

## Graphical Abstract

**Graphical Abstract:**
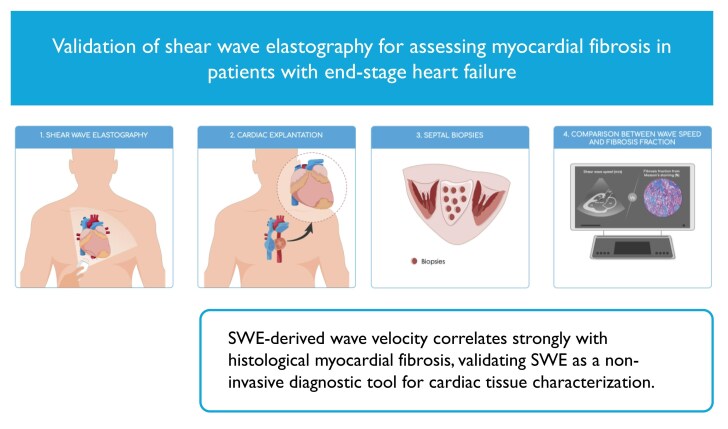
Workflow of the study. (1) Shear wave elastography was performed to measure shear wave velocities. (2) Cardiac explantation was conducted. (2) Septal biopsies were taken from the explanted heart for histological analysis. (4) Shear wave speeds were compared with collagen volume fraction (CVF).

**See the editorial comment for this article ‘Measuring myocardial stiffness: when natural waves play tricks on us’, by M. Venet and O. Villemain, https://doi.org/10.1093/ehjci/jeag042.**

## Introduction

Myocardial fibrosis is a central component of cardiac remodelling.^1^ It leads to cardiac stiffening,^2^ which is fundamental to the pathophysiology of heart failure.^3^ Increased left ventricular (LV) stiffness results in reduced compliance, abnormal LV filling and/or increased filling pressures.^4^ While cardiac imaging techniques traditionally provide structural and functional assessment of the LV, they only provide indirect information about its underlying mechanical properties.^5^ In contrast, measurement of cardiac stiffness may provide direct insight into the fibrotic remodelling of the heart, offering an opportunity to refine the diagnostic accuracy of cardiac diseases and lead to better patient management and improved prognosis.^6^ Although cardiac catheterization is an accurate method for measuring LV chamber stiffness, the invasive nature and associated risks limit its routine use in clinical practice.^7^ Non-invasive measurement of myocardial stiffness directly is currently lacking in clinical care.^8^

Shear wave elastography (SWE) is an emerging non-invasive imaging technique which may permit quantification of myocardial stiffness and fibrosis.^9^ This technique uses high frame rate echocardiography to measure the velocity of artificially induced or naturally occurring shear waves in the myocardium. The propagation speed of shear waves is directly related to tissue stiffness and may therefore reflect the fibrotic development in the heart.^10^ However, the ability of SWE to assess myocardial fibrosis remains to be validated histologically.

The purpose of this study was to validate SWE for assessing myocardial fibrosis in the failing human heart. We aimed to compare naturally occurring shear wave velocities in patients scheduled for heart transplantation with collagen volume fraction (CVF) as measured by histology after explantation.

## Methods

### Study population

Oslo University Hospital Rikshospitalet performs all solid organ transplantations in Norway. We prospectively enrolled patients who were referred for evaluation for heart transplantation and patients who were already on the heart transplant waiting list. The workflow of the study is illustrated in the Central figure. Patients were included irrespective of aetiology of heart failure and irrespective of whether they had cardiac implantable electronic devices (CIED), LV assist devices (LVAD) or implanted prosthetic valves. We also recruited healthy sex- and age matched controls who had no family history of hereditary cardiomyopathy, no signs or symptoms of chronic disease, normal blood pressure (<140/90 mm Hg), no use of prescription drugs, a body mass index < 30 kg/m^2^, and no ongoing pregnancy. The study was conducted according to the principles of the Declaration of Helsinki and by approval of the Regional Committee for Medical and Health Research Ethics in South-Eastern Norway (approval numbers: 230524, 434476, and 584637). Written informed consent was obtained from all study participants.

### Echocardiography

We performed two-dimensional transthoracic echocardiography according to current recommendations^11^ using a Vivid E95 ultrasound system and an M5Sc-D 1.5–4.6 MHz phased array transducer (GE Vingmed Ultrasound, Horten, Norway). Images were analyzed offline using GE EchoPAC version 206. Evaluation of LV filling pressure was performed according to current recommendations.^12,13^

### High frame rate imaging

High frame rate imaging and analysis was performed by the same cardiologist (KA) for all patients using the same commercially available hardware as for the conventional echocardiography. The healthy controls were either examined by the same cardiologist or certified sonographers with training in SWE. The left ventricle was imaged from the parasternal long-axis view (PLAX), as well as the apical four-chamber (A4C) and APLAX views. High frame rate imaging was performed by transmitting diverging waves with a focus of −2.5 cm in azimuth and 8.6 cm in elevation to be processed by second harmonic imaging and retrospective transmit beamforming. A frame rate of 1199 Hz was obtained by compounding four diverging waves using a default sector width of 65° and depth of 13 cm, influenced only by the need of the operator to adjust the scan area. Two thousand frames of in-phase and quadrature component (IQ) data per view were extracted and stored offline for analysis.

### Shear wave analysis

**Figure 1:**
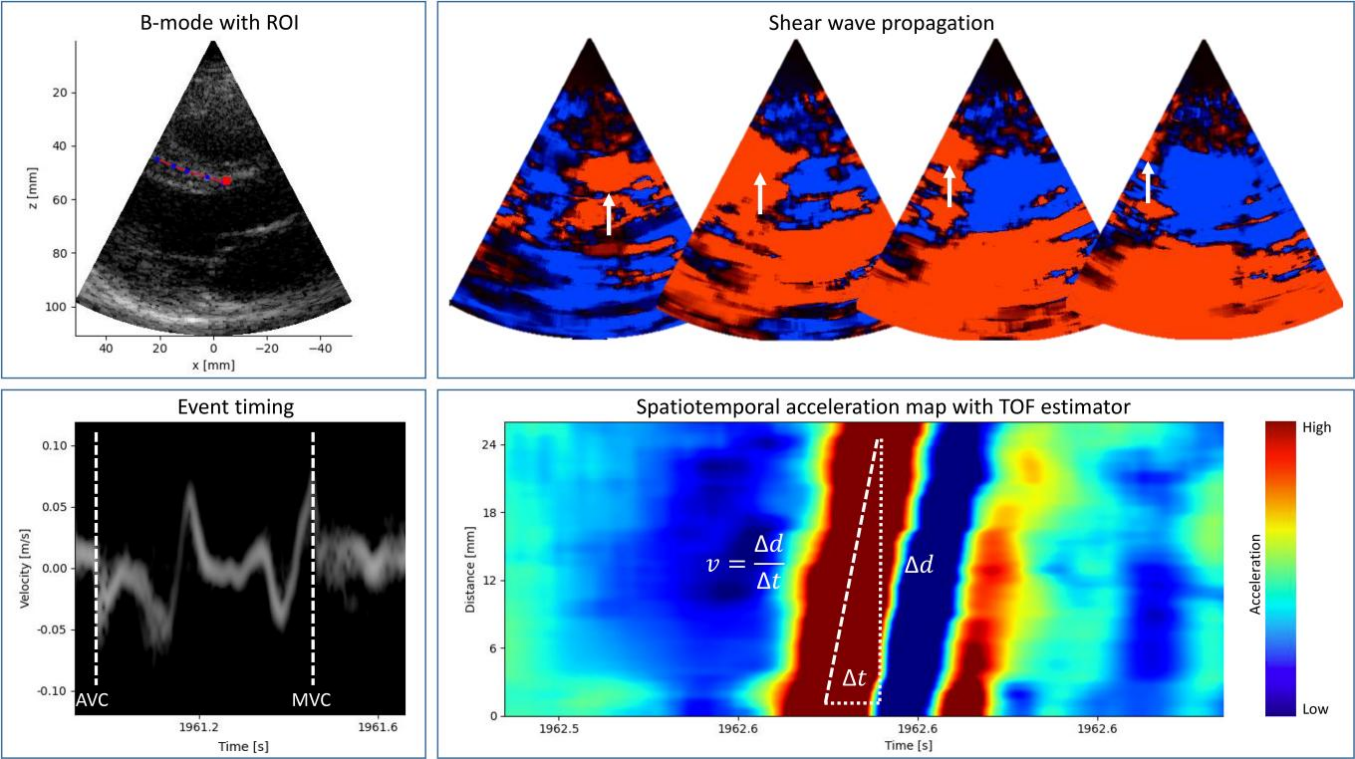
SWE. Top left panel: B-mode image from a PLAX with ROI in the IVS (dashed line) and marker for pulsed tissue Doppler in the basal septum. Top right panel: Analysis of the raw IQ data by estimating tissue motion by tissue Doppler imaging. White arrows follow an AVC wave propagating in the IVS. Bottom left panel: Tissue Doppler curve for identifying shear waves triggered by intrinsic events in the cardiac cycle. Bottom right panel: Spatiotemporal acceleration map measuring shear wave velocity by application of a time-of-flight estimator. AVC, aortic valve closure; IQ, in-phase and quadrature data components; MVC, mitral valve closure; ROI, Region of interest; TOF, time-of-flight.

Shear wave analysis is illustrated in *Figure 1*. We analyzed the IQ data using custom software (PyMWI)^14^ to visualize shear wave propagation in the interventricular septum (IVS) by tissue Doppler imaging. Shear waves were identified and tracked by estimating tissue motion derived from the IQ data by the lag-one autocorrelation of the temporal signals.^15^ Smoothing parameters for the tissue-Doppler images were set to 8 × 8 image points (depth × width), and a uniform (box) forward-backward filter corresponding to 5 ms in time, both applied on the tissue-Doppler autocorrelation estimates. Acceleration traces were calculated by taking the first order derivative of the TDI time series. Spatiotemporal acceleration maps were obtained by placing an anatomical M-mode line from base to apex in the region of interest (ROI) of the B-mode images, performing additional spatial smoothing along the anatomical M-mode line using 5 mm in the transversal and 2 mm in the longitudinal direction. Shear wave position was represented as a function of time. The ROI was selected as the mid-myocardium of the IVS, excluding the annular plane and the apical cap. The inferior septum was assessed from the A4C view and the anterior septum from PLAX and APLAX views.^11,16^ Gain settings were adjusted manually for optimal shear wave visualization.

Intrinsic shear waves generated by the mitral valve closure (MVC) and aortic valve closure (AVC), throughout this paper referred to as MVC waves and AVC waves, were identified by comparing the shear wave position in the spatiotemporal acceleration map to the mitral annular velocity trace by tissue Doppler and the valve closure in the B-mode images. The propagation speed of the shear waves was then calculated using a time-of-flight estimator.^17^ Shear wave images of insufficient quality as based on previously proposed criteria^14^ were excluded from the analysis, discarding waves with unphysiologic velocities, bidirectional propagation or of poor image quality. Shear waves with velocities exceeding 20 m/s were excluded from the analysis as this is the upper limit of expected shear wave propagation speed in myocardial tissue.^17^ We measured overall septal shear wave propagation speed from the PLAX, A4C, and APLAX views. Parasternal and apical measurements were analyzed separately as direct comparison of these imaging planes have shown differences in shear wave speeds with poor correlation between measurements.^18^ Regional analyses were performed separately by visually dividing the IVS into basal, mid and apical regions according to current recommendations for LV segmentation.^11^ Values are reported as mean of measurements per wave from non-excluded data.

### Tissue sampling and histological analysis

**Figure 2:**
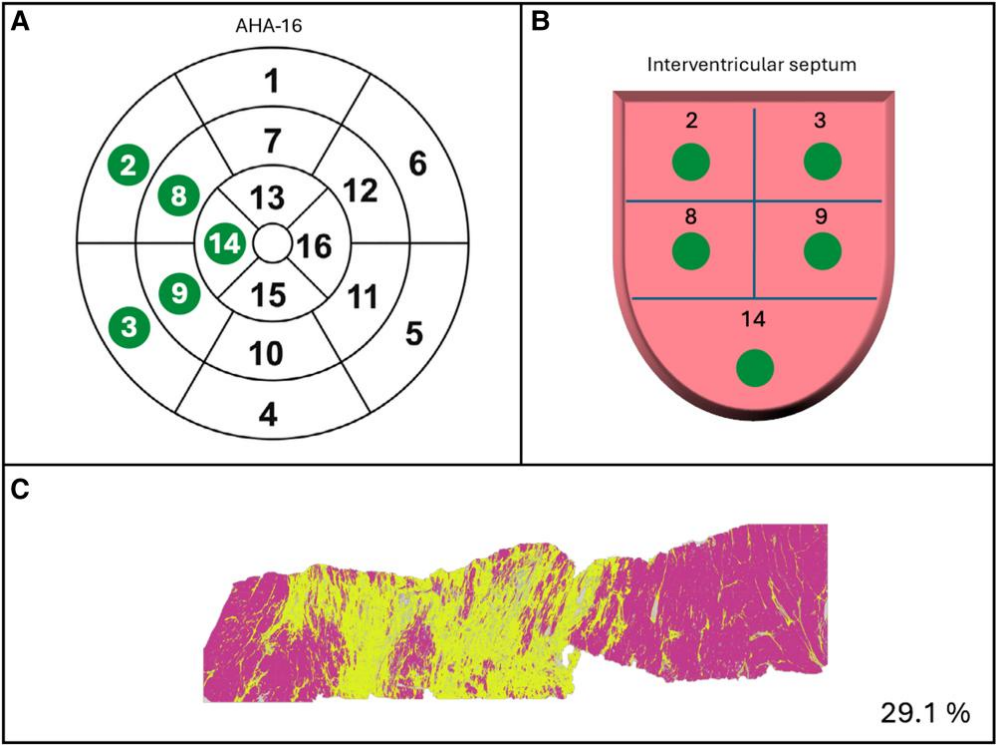
Biopsy schematic. (A) Septal segments include segment 2, 3, 8, 9 and 14. 2: basal anteroseptal, 3: basal inferoseptal, 8: mid-ventricular anteroseptal, 9: mid-ventricular inferoseptal, 14: apical septal. The coloured dots represent biopsies. (B) Schematic of the IVS. (C) Example of transmural biopsy which has been analyzed with a classifier. Yellow is fibrosis and pink is not fibrosis. CVF in this example is 29.1%.

Myocardial tissue samples were obtained from the patients shortly after cardiac explantation {median time from explantation to first taken sample was 43 [interquartile range (IQR) 30–55] minutes}. The cardiac explants were dissected into the left ventricle, IVS and right ventricle, and were maintained in ice-cold solution when not undergoing the sampling procedure. Samples were organized according to the American Heart Association's 16-segment model (AHA16),^16^ enabling precise comparison between localized imaging findings and corresponding histological data (*Figure 2*). In the apical septal region (segment 14), two samples were obtained and averaged to yield a single CVF for that region. The samples were cylindrical in shape (6–8 mm diameter) and encompassed the entire transmural depth of the myocardium. Samples were embedded in optimal cutting temperature compound, before they were frozen in liquid nitrogen and subsequently stored in a −80°C freezer. They were sectioned in the transmural direction and stained with Masson's trichrome for histological quantification of fibrosis. Fibrosis was quantified by calculating the percentage of fibrotic tissue in each sample with QuPath version 0.4.3.

For this study, we used only the septal samples. Mean septal CVF was obtained from averaging the CVF of all available septal samples for each patient. Regional septal CVF refers to the CVF from each individual sample defined by the AHA16 model (*Figure 2*). For comparative purposes, an upper limit of normality for septal CVF of 2.1% was chosen based on historical data.^19^

### Statistical analysis

Descriptive statistics are reported as mean ± standard deviation (SD), median (IQR) or numbers (%) as appropriate. We used independent samples *T*-test and Mann–Whitney *U* test to evaluate between-group differences for continuous variables and Fischer’s Exact test for categorical data. Associations between shear wave velocities and mean septal CVF were analyzed using Spearman’s correlation. For regional analysis, we used linear mixed-effects regression models taking within-subject correlation into account. We performed Bland-Altman analysis^20^ to assess intra- and interobserver reproducibility for the control subjects as several operators had been involved in the image acquisition for this group. Statistical significance was set at a *P*-value of ≤0.05. Analyses were performed using STATA v18.0.

### Data management

All data were deidentified and securely stored in compliance with data protection regulations. Data analysis was performed in a secure computing environment.

## Results

### Study population

From October 2022 to March 2025, a total of 16 heart failure patients who had been investigated by SWE underwent heart transplantation. Median time span from SWE to heart transplantation was 113 (15–151) days. We report baseline characteristics for heart failure patients and controls in *Table 1*.

**Table 1:** Baseline characteristics of heart failure patients and controls

	Heart failure	*n*	Control	*n*
Age, years	48 ± 15	16	48 ± 15	16
Male, *n*	13 (81%)	16	13 (81%)	16
SBP, mmHg	101 ± 11	11	134 ± 10	16
DBP, mmHg	67 ± 11	11	81 ± 9	16
Heart rate, bpm	75 ± 12	16	62 ± 12	16
BMI, kg/m^2^	27 ± 4	16	25 ± 4	16
Atrial fibrillation, *n*	3 (19%)	16	0 (0%)	16
NYHA class				
II, *n*	3 (21%)	14	—	—
III, *n*	10 (71%)	14	—	—
IV, *n*	1 (7%)	14	—	—
VO_2max_, mL/kg/min	14 ± 4	14	—	—
Type of heart failure				
HFrEF, *n*	15 (94%)	16	—	—
HFmrEF, *n*	0 (0%)	16	—	—
HFpEF, *n*	1 (6%)	16	—	—
Aetiology				
DCM, *n*	7 (44%)	16	—	—
HCM, *n*	3 (19%)	16	—	—
ARVC, *n*	2 (13%)	16	—	—
Sarcoidosis, *n*	2 (13%)	16	—	—
Ischemic heart disease, *n*	1 (6%)	16	—	—
Congenital heart disease, *n*	1 (6%)	16	—	—
CIED, *n*	13 (81%)	16	—	—
CRT-D, *n*	9 (56%)	16	—	—
ICD, *n*	3 (19%)	16	—	—
Pacemaker, *n*	1 (6%)	16	—	—
LVAD, *n*	4 (25%)	16	—	—
Prosthetic valve, *n*	3 (19%)	16	—	—
Mechanical AVR, *n*	1 (6%)	16	—	—
Mechanical MVR, *n*	1 (6%)	16	—	—
Biological MVR, *n*	1 (6%)	16	—	—
Lab results				
Hemoglobin, g/L	141 ± 17	16	147 ± 11	16
CRP, mg/L	6 ± 5	16	1 ± 0	16
Creatinine, µmol/L	114 ± 42	16	80 ± 15	16
NT-proBNP, ng/L	3055 ± 4224	13	53 ± 8	16
Troponin T, ng/L	48 ± 53	13	7 ± 5	16

Data are shown as mean ± SD or *n* (%).

ARVC, arrhythmogenic right ventricular cardiomyopathy; AVR, aortic valve replacement; BMI, body mass index; BPM, beats per minute; CIED, cardiac implantable electronic device; CRP, C-reactive protein; CRT-D, cardiac resynchronization therapy defibrillator; DBP, diastolic blood pressure; DCM, dilated cardiomyopathy; HCM, hypertrophic cardiomyopathy; HFmrEF, heart failure with mid-range ejection fraction; HFpEF, heart failure with preserved ejection fraction; HFrEF, heart failure with reduced ejection fraction; ICD, implantable cardioverter defibrillator; LVAD, left ventricular assist device; MVR, mitral valve replacement; NT-proBNP, *n*-terminal pro-B type natriuretic peptide; NYHA, New York Heart Association; SBP, systolic blood pressure; VO2max, maximum oxygen consumption.

### Septal fibrosis

**Figure 3:**
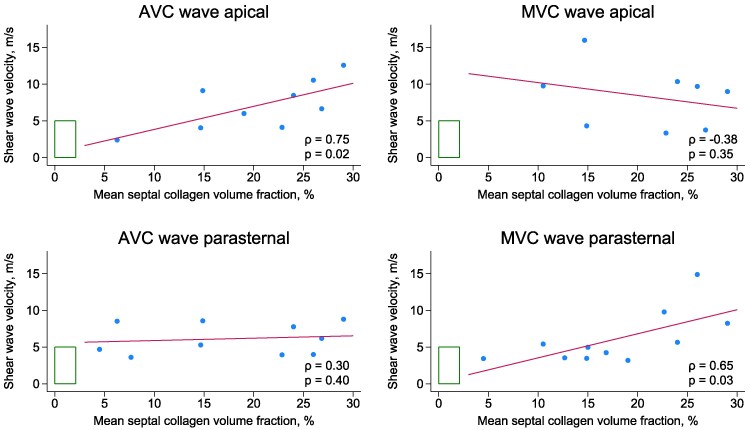
Correlations between mean septal CVF and septal shear wave velocities by imaging plane. Each dot represents mean values for a unique patient, and the line shows the result of linear regression. Green area indicates expected upper ranges of normality in healthy individuals for CVF^19^ and shear wave velocities.^9^ AVC, aortic valve closure; MVC, mitral valve closure.

**Figure 4:**
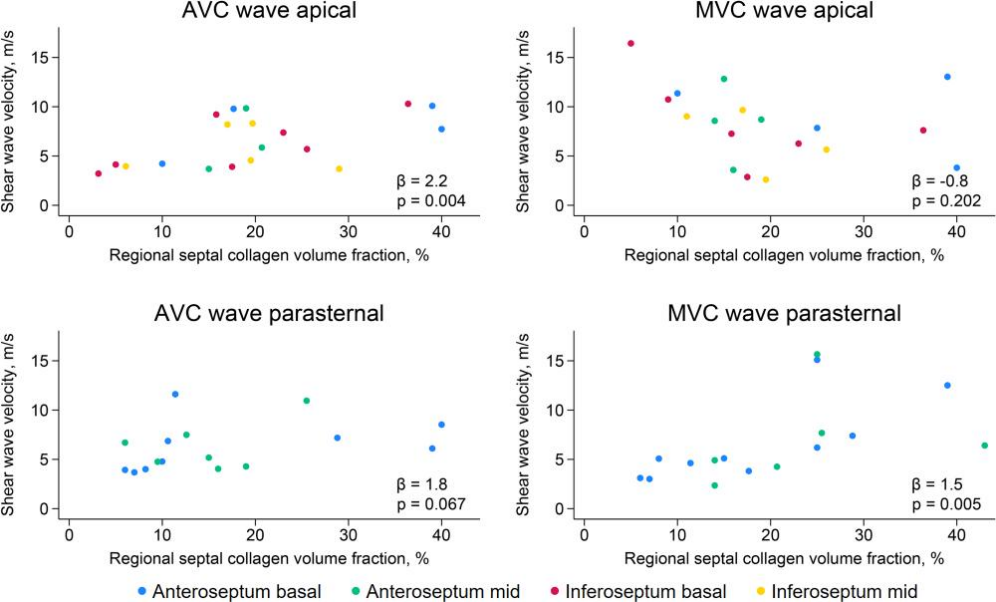
Scatterplots of regional septal shear wave velocities and regional septal CVF colour-coded by septal region. Each dot represents septal shear wave velocities and CVF in the corresponding segment for a unique patient. Analyses are performed separately for MVC/AVC waves and by apical/parasternal imaging views. Regression coefficients (*β*) and *P*-values are reported from linear mixed-effects regression models. AVC, aortic valve closure; MVC, mitral valve closure.

The mean septal CVF was 17.1 ± 7.6%. The basal, middle and apical regions had mean CVF of 18.5 ± 9.6%, 15.1 ± 9.0% and 17.8 ± 10.7%, respectively. The association between septal CVF and corresponding shear wave velocities is shown in *Table 2*. Mean septal CVF was strongly associated with AVC wave velocities from apical views (*ρ* = 0.75, *P* = 0.02) and moderately associated with MVC wave velocities from the PLAX (*ρ* = 0.65, *P* = 0.03) (*Figure 3*). Similar results were observed in regional analysis where both septal AVC wave and MVC wave velocities were associated with septal CVF in the corresponding region by linear mixed-effects regression (*Table 2* and *Figure 4*).

**Table 2:** Associations between septal shear wave velocities and septal CVF

Shear wave	Mean septal CVF%			Regional septal CVF%			
	*N*	*P*	*P*-value	*N*	*β*	95% CI	*P*-value
AVC wave							
Apical	9	0.75	0.02	19	2.2	0.7, 3.8	0.004
Parasternal	10	0.30	0.40	16	1.8	−0.1, 3.7	0.067
MVC wave							
Apical	8	−0.38	0.35	18	−0.8	−2.0, 0.4	0.202
Parasternal	11	0.65	0.03	16	1.5	0.4, 2.5	0.005

Mean of septal values were analyzed by Spearman’s correlation and regional septal values by linear mixed-effects regression models to account for within-subject correlation.

AVC, aortic valve closure; CVF, collagen volume fraction; MVC, mitral valve closure; *ρ*, Spearman’s Rho; *β*, regression coefficient.

### Shear wave velocities and feasibility

Overall measurements indicate faster shear wave velocities in patients with heart failure than in controls, except for apical measurements for the MVC wave (*Table 3*). We report results by region in Supplementary data online, *Table S1* and feasibility of shear wave analysis in Supplementary data online, *Table S2*. In most patients, reliable shear wave traces could not be obtained for the apical region, which we therefore excluded from the remaining SWE analyses.

**Table 3:** Shear wave velocities in the IVS for patients with end-stage heart failure and control subjects

Shear wave	MVC wave			AVC wave		
	Heart failure	Controls	*P*-value	Heart failure	Controls	*P*-value
PLAX (m/s)	6.1 ± 3.6	3.8 ± 1.1	0.02	6.1 ± 2.1	4.1 ± 1.4	0.007
A4C (m/s)	8.4 ± 4.0	7.7 ± 3.3	0.78	6.1 ± 3.8	5.1 ± 1.7	0.41
APLAX (m/s)	8.1 ± 4.4	7.6 ± 2.2	0.77	7.6 ± 2.8	4.4 ± 1.3	0.002
Mean apical (m/s)	8.3 ± 4.3	7.8 ± 0.7	0.78	7.1 ± 3.3	4.7 ± 1.1	0.02

*P*-values for comparison of shear wave velocities between groups. Sample sizes are shown in Supplementary data online, *Table S2*.

A4C, apical four-chamber; APLAX, apical long-axis; AVC, aortic valve closure; IVS, interventricular septum; MVC, mitral valve closure; PLAX, parasternal long-axis.

### Left ventricular structure and function

Compared with controls, heart failure patients had larger LV end-systolic volumes and poorer LV systolic function (see Supplementary data online, *Table S3*). There was no difference in interventricular septal thickness. Lower mitral annular tissue velocities, higher E/e′ ratio and larger left atrial volumes in heart failure patients suggest impaired diastolic function. Thirty-eight percent of the heart failure patients had estimated normal LV filling pressures compared with 100% of controls.

### Reproducibility

**Figure 5:**
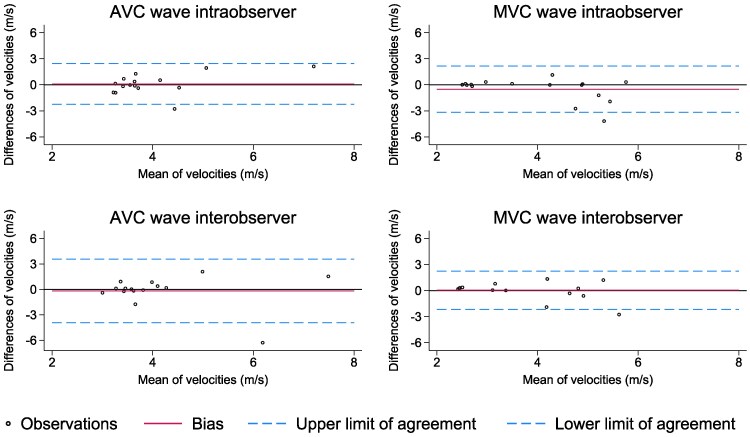
Bland-altman plots of intra- and interobserver reproducibility of SWE from a PLAX view in the control subjects. There was no bias for either intra- or interobserver analyses. AVC, aortic valve closure; MVC, mitral valve closure; PLAX, parasternal long-axis; SWE, shear wave elastography.

There was no indication of bias in intra- or interobserver analyses by Bland-Altman plots (*Figure 5*). Bias (limits of agreement) for intraobserver analyses were 0.1 (−2.3, 2.5) m/s and −0.5 (−3.2, 2.2) m/s for AVC and MVC waves, respectively, whereas for interobserver analyses the corresponding results were −0.2 (−3.9, 3.6) m/s and 0.0 (−2.2, 2.2) m/s.

## Discussion

This is the first study to validate SWE by high frame rate echocardiography against histological assessment of myocardial fibrosis. The main results of our study are: (1) shear wave velocities that occur naturally after AVC and MVC correlate to CVF in the human heart; and (2) SWE can be successfully performed in patients with end-stage heart failure across diverse aetiologies.

### Shear wave velocities and myocardial fibrosis

We found that both septal AVC wave and MVC wave velocities correlate with myocardial fibrosis assessed as septal CVF. The association between CVF and AVC wave velocities was strong, whereas the association to MVC wave velocities was moderate. Both associations remained significant also when comparing regional shear wave velocities to the CVF of the corresponding region. The former implies that SWE has the potential to quantify overall degree of myocardial fibrosis, whereas the latter may imply that SWE also has the potential to detect regional disease which can further enhance the diagnostic utility of this method.

Although it has been hypothesized that shear wave velocities increase with increased myocardial fibrosis,^9^ our study is the first to histologically validate this hypothesis in a clinical population. Our findings are in line with results from cardiac magnetic resonance (CMR) imaging studies, in which high shear wave velocities have been associated with longer native T1 time and the presence of late gadolinium enhancement (LGE),^21–23^ both of which are established biomarkers of myocardial fibrosis.^24,25^ Myocardial fibrosis by CMR is associated with morbidity and mortality in clinical populations,^26^ but routine use of CMR in clinical practice is limited by availability, long exam durations and cardiac implantable devices.^27^ This leaves a need for novel methods for non-invasive myocardial tissue characterization which, based on the results from our study, may be met by SWE.

### Shear wave velocities and imaging plane

Our results demonstrate associations between shear wave velocities and myocardial fibrosis in heart failure patients, which was evident in apical views for AVC waves and from a parasternal view for MVC waves. The association between shear wave velocities and myocardial fibrosis hence seems to be dependent on the imaging plane. The most studied natural shear wave in SWE is the MVC wave from a PLAX view, where our results support previous indirect data from CMR indicating an association between MVC wave velocities and myocardial fibrosis.^21,23^ As for AVC waves, only one study have investigated the potential association between CMR biomarkers of myocardial fibrosis and shear wave velocities from a PLAX view, without demonstrating such an association.^14^ These results are in line with the lack of association between myocardial fibrosis and AVC wave velocities from the PLAX view in our study. No previous data on CMR markers of myocardial fibrosis and shear wave velocities from apical views have been reported.

These differences in results might be explained by different components of the shear wave being measured depending on the imaging plane. In elasticity imaging, soft tissue is often approximated as an infinite linear elastic isotropic incompressible material in which a linear relationship between the applied stress and imposed strain describes the material stiffness.^28^ Theoretically only two modes of waves can exist in such a medium, compressional waves and shear waves, where the propagation speed of both wave modes are directly related to tissue stiffness.^17^ Whereas compressional waves used for conventional echocardiography have particle motion longitudinal to the direction of wave propagation, shear waves used for SWE have particle motion transversal to the wave propagation direction.^17^ Due to the position of the IVS, SWE from the parasternal view assesses the transverse component of wave propagation, whereas apical views record the longitudinal component of the wave.^9^ This implies that parasternal imaging theoretically should be preferred for SWE of the IVS, as tissue Doppler imaging is most sensitive to particle motion along the direction of the ultrasound beam.^18^ However, in cardiac SWE, several factors violate these approximations as myocardial stiffness will also depend on fibre orientation, deformation rate and state of myocardial stretch and contraction.^9^ Myocardial tissue is hence more correctly described as a finite non-linear viscoelastic anisotropic medium, in which both transversal and longitudinal wave motion might be evoked.^17^ The presence of both transversal and longitudinal wave components might explain why intrinsic shear waves can be detected from both parasternal and apical imaging views. However, direct comparison of parasternal and apical views in SWE has shown differences in shear wave speeds with poor correlation between measurements,^18^ hence these results cannot be used interchangeably. This aligns with our results where the association between shear wave speeds and myocardial fibrosis was clearly dependent on the imaging plane.

Our results indicate that in the assessment of myocardial fibrosis by SWE, parasternal imaging may be preferred for MVC waves, whereas apical imaging may be best suited for AVC waves.

### Shear wave velocities and myocardial stiffness

SWE offers a direct and non-invasive measurement of the mechanical properties of the myocardium by measuring the speed of shear wave propagation in the heart muscle. The velocity at which these waves travel is closely related to myocardial stiffness in experimental animal studies.^9,29–31^ Although the propagation speed of shear waves is expected to increase with increasing tissue stiffness,^17^ the conversion from velocity to other stiffness constants, such as shear modulus, depends on several assumptions and is not recommended.^9^ We therefore report propagation speed as the preferred metric for analyzing shear waves in our study.

### Other factors potentially affecting shear wave velocities

Our results demonstrate moderate-to-strong associations between increasing shear wave velocities and myocardial fibrosis. However, in addition to pathology, several other factors may also affect shear wave velocities including LV geometry,^32^ contractility,^33^ loading conditions such as preload^21,29,34^ and afterload,^29^ myocardial fibre orientation,^35^ imaging plane^18^ and age.^36,37^ We cannot rule out the potential effects of such factors on our results as the limited sample size in our study does not permit more advanced statistical modelling to adjust for potential confounders. We have employed the gold standard for assessing myocardial fibrosis by histological analysis of the explanted human heart and we perceive our results on the association between shear wave velocities and myocardial fibrosis to be valid. However, expanding further to measure myocardial stiffness by SWE, if possible, would require incorporation of all the above-mentioned factors in a comprehensive model, which was beyond the scope of this study.

### Feasibility of SWE

SWE for the measurement of septal shear wave velocities was successfully conducted in most patients in our study despite the diverse population of patients, and regardless of acoustic quality. This suggests that the method can give important information in the assessment of patients with diverse cardiac diseases. Feasibility of shear wave analysis in our study did however seem to be dependent on shear wave category and imaging plane. For the AVC wave, most of the unsuccessful analyses were explained by intrinsic patient factors such as prosthetic valves and/or LVADs. This also highlights the limitations of relying on naturally occurring events in the cardiac cycle for generation of shear waves. Analysis of MVC and AVC waves assumes that there is a substantial transmission of shear waves from valvular closure to the myocardium, which is violated in the case of LVADs and prosthetic valves. Degenerative valve disease may also limit this methodology, as demonstrated by relatively poor feasibility for evaluating AVC waves in severe aortic stenosis.^14^ As for MVC waves, feasibility seemed adequate for parasternal images, but needs to be improved for apical images for this to have any diagnostic utility.

## Limitations

Patients were examined by SWE at the time of evaluation for heart transplantation, whereas histological analyses could not be performed until after the patients’ heart had been explanted. Performing SWE at the time of transplantation was not considered ethical as this would further complicate the transplant logistics and introduce additional risk to the recipients. All patients listed for heart transplantation at our centre are routinely re-evaluated at 3-to-4-months’ time intervals to detect potential deterioration of their clinical condition or other intercurrent disease. However, disease progression or intercurrent events in the time span from SWE to heart transplantation may have introduced random variability in our material.

This is a single-centre study which limits the external validity of our results. Currently, no commercially available software for SWE exists in cardiology, which is a prerequisite for broader expansion of the method. Our sample size is relatively small, leaving the results vulnerable to type-2 errors and susceptible to outliers, and this also precludes more advanced statistical modelling such as multivariable analyses to adjust for potential confounders. The low number of patients listed for heart transplantation limits sample sizes for single-centre studies. Despite promising results demonstrating moderate-to-strong associations between shear wave velocities and CVF, the robustness of our results would be strengthened by confirmation in larger studies applying standardized equipment and multi-centre design.

## Conclusion

Septal shear wave velocities after aortic and MVC correlate with CVF. Our findings therefore suggest that SWE can be used to assess myocardial fibrosis. This technique holds the potential to become a novel cornerstone in non-invasive cardiac imaging.

## Supplementary Material

jeaf375_Supplementary_Data

## Data Availability

Data may be available upon reasonable request to the authors.
