# Supplementary material for: Validation of shear wave elastography for assessing myocardial fibrosis in patients with end-stage heart failure
Source: Eur Heart J Cardiovasc Imaging. 2026 Jan 6;27(4):778–87. doi: 10.1093/ehjci/jeaf375 (PMC13021274; doi:10.1093/ehjci/jeaf375)
Supplement: jeaf375_Supplementary_Data [file jeaf375_supplementary_data.docx]

**Supplementary materials**

|  | **MVC wave** | | | **AVC wave** | | | |
| --- | --- | --- | --- | --- | --- | --- | --- |
|  | Heart failure | Control | p-value | Heart failure | Control | p-value |  |
| **PLAX** |  |  |  |  |  |  |  |
| Basal (m/s) | 6.3±4.0 | 3.7±1.2 | 0.02 | 6.5±2.5 | 3.9±1.0 | 0.002 |  |
| Mid (m/s) | 6.6±4.4 | 4.3±1.3 | 0.19 | 6.0±2.4 | 4.5±0.9 | 0.06 |  |
| **A4C** |  |  |  |  |  |  |  |
| Basal (m/s) | 8.3±4.3 | 9.9±4.9 | 0.54 | 5.8±2.9 | 5.2±1.8 | 0.55 |  |
| Mid (m/s) | 7.7±3.3 | 7.0±4.5 | 0.77 | 5.9±2.3 | 3.7±1.6 | 0.05 |  |
| **APLAX** |  |  |  |  |  |  |  |
| Basal (m/s) | 7.5±3.7 | 6.0±1.6 | 0.34 | 7.9±2.7 | 4.1±1.3 | 0.0004 |  |
| Mid (m/s) | 8.8±3.0 | 5.8±1.7 | 0.02 | 6.7±2.3 | 4.0±1.7 | 0.02 |  |

Supplementary table 1: Regional shear wave velocities in the interventricular septum of end-stage heart failure patients and controls. P-values for comparison of shear wave velocities between groups. Sample sizes are shown in Supplementary table 2. A4C: apical four-chamber. APLAX: apical long-axis. AVC: aortic valve closure. MVC: mitral valve closure. PLAX: parasternal long-axis.

|  | Heart failure (n=16) | | Control (n=16) | |
| --- | --- | --- | --- | --- |
|  | MVC wave | AVC wave | MVC wave | AVC wave |
| **PLAX** | **11 (69%)** | **10 (63%)** | **16 (100%)** | **15 (94%)** |
| Basal | 11 (69%) | 10 (63%) | 15 (94%) | 15 (94%) |
| Mid | 7 (44%) | 8 (50%) | 9 (56%) | 13 (81%) |
| **A4C** | **6 (38%)** | **6 (38%)** | **4 (25%)** | **14 (88%)** |
| Basal | 7 (44%) | 6 (38%) | 6 (38%) | 13 (82%) |
| Mid | 6 (38%) | 5 (31%) | 5 (31%) | 10 (63%) |
| Apical | 2 (13%) | 3 (19%) | 3 (19%) | 3 (19%) |
| **APLAX** | **6 (38%)** | **8 (50%)** | **10 (63%)** | **13 (81%)** |
| Basal | 7 (44%) | 7 (44%) | 7 (44%) | 13 (81%) |
| Mid | 6 (38%) | 7 (44%) | 9 (56%) | 8 (50%) |
| Apical | 3 (19%) | 4 (25%) | 2 (13%) | 0 (0%) |

Supplementary table 2: Feasibility of shear wave elastography in the interventricular septum. Number of successful assessments for each view, evaluated in the entire septum (bold) and by region (regular formatting). A4C: apical four-chamber. APLAX: apical long-axis. AVC: aortic valve closure. MVC: mitral valve closure. PLAX: parasternal long-axis.

| **Parameter** | **Heart failure** | **n** | **Control** | **n** | **P-value** |
| --- | --- | --- | --- | --- | --- |
| IVSd | 9±3 | 16 | 8±2 | 16 | 0.63 |
| LVEDVi, ml/m^2^ | 91 (67-115) | 16 | 74 (64-78) | 16 | 0.06 |
| LVESVi, ml/m^2^ | 69 (45-88) | 16 | 29 (27-33) | 16 | <0.0001 |
| LVEF, % | 29±9 | 16 | 58±3 | 16 | <0.0001 |
| LVGLS, % | -7±3 | 15 | -18±1 | 16 | <0.0001 |
| LAVi, ml/m^2^ | 49±21 | 13 | 31±8 | 16 | 0.003 |
| LASr, % | 11±6 | 14 | 28±6 | 15 | <0.0001 |
| e' septal, m/s | 0.04±0.01 | 12 | 0.09±0.03 | 16 | <0.0001 |
| e' lateral, m/s | 0.07±0.02 | 12 | 0.12±0.04 | 16 | 0.0002 |
| E/A ratio | 2.15±1.92 | 12 | 1.42±0.43 | 15 | 0.17 |
| E/e' septal | 18.67±11.04 | 12 | 6.78±1.70 | 15 | 0.0004 |
| E/e' lateral | 10.14±5.47 | 12 | 4.75±1.33 | 15 | 0.001 |
| TRP, mm Hg | 23±13 | 14 | 19±3 | 15 | 0.24 |
| Estimated filling pressure |  | 16 |  | 16 | 0.001 |
| Normal, n | 6 (38%) |  | 16 (100%) |  |  |
| Increased, n | 4 (25%) |  | 0 (0%) |  |  |
| Indeterminate, n | 6 (38%) |  | 0 (0%) |  |  |

Supplementary table 3 – Echocardiography characteristics of end-stage heart failure patients and controls. IVSd: interventricular septal diastolic thickness. LAVi: indexed left atrial volume. LASr: left atrial reservoir strain. LVEDVi: indexed left ventricular end diastolic volume. LVEF: left ventricular ejection fraction. LVGLS: left ventricular global longitudinal strain. LVESVi: indexed left ventricular end systolic volume. TRP: tricuspid regurgitation pressure.
